# Prognostic Impact of UBA1 Expression in Breast Cancer

**DOI:** 10.7150/jca.129539

**Published:** 2026-03-17

**Authors:** Jana Koch, Lea Vatter, Jannis Heyer, Maximilian Klar, Ingolf Juhasz-Böss, Martin Werner, Konrad Kurowski, Peter Bronsert

**Affiliations:** 1 Institute of Surgical Pathology, Medical Centre - University of Freiburg, Freiburg, Germany.; 2 Faculty of Medicine, University of Freiburg, Germany.; 3 German Cancer Consortium (DKTK) and Cancer Research Centre (DKFZ), Heidelberg, Germany.; 4 Tumorbank Comprehensive Cancer Centre Freiburg, Medical Centre - University of Freiburg, Germany.; 5 Core Facility for Histopathology and Digital Pathology, Medical Centre - University of Freiburg, Germany.; 6 Department of Obstetrics & Gynaecology Medical Centre, University of Freiburg, Germany.; 7 Department of Obstetrics & Gynaecology, Kantonsspital Aarau, Switzerland.

**Keywords:** breast cancer, UBA1, prognostic factor, AI-assisted analysis, digital pathology

## Abstract

**Background:**

The use of established prognostic markers has improved the diagnostic stratification and therapeutic approaches of breast cancer. Ubiquitin-like modifier activating enzyme 1 (UBA1), a key enzyme in the ubiquitin-proteasome pathway, has been reported to play a role in the pathogenesis of various malignant tumors. However, its functional impact on breast cancer progression, especially across intrinsic subtypes, remains poorly understood. This study aimed to evaluate the prognostic relevance of UBA1 protein expression in patients with breast cancer.

**Methods:**

Formalin-fixed, paraffin-embedded (FFPE) tissue samples from 413 chemotherapy-naïve patients with invasive breast cancer were obtained from the Institute of Surgical Pathology (ISP) at the University Medical Centre Freiburg in Germany. Haematoxylin and eosin (H&E)-stained slides were digitised and annotated to define regions of interest (ROIs) for tissue microarray (TMA) construction. TMA sections were immunohistochemically stained for UBA1 expression and intrinsic subtype markers, including oestrogen receptor (ER), progesterone receptor (PgR), human epidermal growth factor receptor 2 (HER2), and Ki-67. All slides were digitised. UBA1 and intrinsic markers were evaluated and analysed using AI-assisted image analysis software (HALO AI). Furthermore, UBA1 expression was analysed separately in both tumor cells and tumor-associated stroma. All results were statistically correlated with clinicopathological data parameters.

**Results:**

High UBA1 expression in both the tumor and stromal compartments was significantly associated with reduced overall survival (OS). Subtype-specific analyses revealed that elevated stromal UBA1 expression, particularly in the cytoplasm, was associated with poorer survival in luminal A and luminal B subtypes. Conversely, increased nuclear UBA1 expression in tumor cells was associated with worse outcomes in the luminal B subtype. Multivariable Cox regression analyses revealed that UBA1 expression in tumor cells was an independent prognostic marker. Furthermore, bivariate analyses revealed that high stromal UBA1 expression was associated with a broader range of adverse clinicopathological parameters, most notably at the cytoplasmic level.

**Conclusion:**

This study highlights the prognostic significance of UBA1 protein expression in breast cancer, thereby demonstrating its potential utility as a diagnostic and therapeutic target.

## Introduction

Breast cancer is the most prevalent malignant neoplasm among women worldwide 1. The integration of prognostic and predictive biomarkers has enabled personalized treatment strategies 2,3. However, the underlying molecular mechanisms of breast cancer development remain incompletely understood, largely owing to its pronounced biological heterogeneity. Moreover, reliably identifying patients at increased risk of treatment failure remains challenging. Thus, further research into biomarkers that contribute to breast cancer progression and that may serve as potential therapeutic targets appears warranted.

UBA1 is a key enzyme of the ubiquitin-proteasome system (UPS), which was originally described by Hershko and Ciechanover 4,5. While the main function of the UPS is proteasomal protein degradation 6, further essential roles have been identified over the years, including the regulation of cellular homeostasis and DNA repair 7-9. Dysregulation of the UPS has been implicated in tumor progression and carcinogenesis 10. In breast cancer, E3 ubiquitin ligases in particular have been characterised as oncogenic drivers 11.

UBA1 has been investigated as a potential therapeutic target in haematological malignancies 12,13 and has been identified as an essential survival gene in triple-negative breast cancer (TNBC) in genome-wide CRISPR/Cas screening 14. Furthermore, Feng et al. emphasised the prognostic role of UBA1 in breast cancer, reporting that elevated UBA1 mRNA expression is associated with tumor progression and poorer OS 15. Additionally, a few preclinical studies have demonstrated the antitumor effects of proteasome inhibitors in breast cancer, highlighting the potential of the UPS as a therapeutic target 16,17.

Nevertheless, the role of UBA1 in breast cancer, especially across intrinsic molecular subtypes, remains poorly characterised. In this study, we used a combination of immunohistochemistry and AI-assisted digital image analysis to computationally analyse immunohistochemically stained and digitised TMAs, evaluating the prognostic significance of UBA1 protein expression and its distribution across intrinsic molecular subtypes of breast cancer.

## Materials and Methods

This retrospective, single-centre study was conducted at the ISP at the University Medical Centre Freiburg, Germany. The study was performed in accordance with the Declaration of Helsinki and approved by the Ethics Committee of the University of Freiburg (protocol number: 22-1472-S1-retro, 26/01/2023). The study cohort initially comprised 413 tumor samples from chemotherapy-naïve patients with invasive breast cancer, all resected surgically between 2011 and 2020 at the Department of Obstetrics and Gynaecology, University Medical Centre Freiburg, Germany. Written informed consent was obtained from all patients prior to inclusion. Clinicopathological data were retrieved from the ISP and the Department of Obstetrics and Gynaecology. The FFPE tumor samples and corresponding H&E-stained slides were obtained from the ISP.

### Clinicopathological Data

Patient data included age at the time of diagnosis, sex and vital status. The following clinicopathological parameters were recorded for all cases: WHO- 18 and UICC-classification 19 comprising histological subtype, pT-, pN-, L-, V-, Pn-classification, resection status, grading according to Elston & Ellis 20, intrinsic subtypes 2,3,21 and patients´ OS.

### Preparation of the Tissue Microarray Construction

Prior to inclusion, tumor-containing H&E-stained slides were reviewed by board-certified pathologists and then digitised using a high-throughput scanner (PANNORAMIC 1000, 3DHistech Kft., Budapest, Hungary). Tumor ROIs were annotated using CaseViewer software (version 2.3.0.99276, 3DHistech Kft.). Three representative tissue cores, each with a diameter of 1 mm, were selected per case. The corresponding FFPE donor blocks were retrieved from the ISP. To ensure the correct and accurate linkage between patient data and tissue samples, all blocks and slides were labelled with barcodes created using Barcode Generator software (Bytescouts, version 7.3). These labels were then printed using a Zebra ZD421 printer (Zebra Technologies Corporation, Lincolnshire, IL, USA).

### Creation of Tissue Microarrays

TMAs were generated using the automated TMA Grand Master (version 3.2.8.125484, 3DHistech Kft., Budapest, Hungary). Tissue cores from FFPE donor blocks were inserted into previously defined positions within acceptor blocks to generate the TMAs. FFPE tumor blocks were processed sequentially and chronologically using the TMA Grand Master microarrayer. A total of 72 blocks were processed in parallel, including 60 donor blocks and 12 acceptor blocks. Digital images of all the blocks were generated using an integrated camera system and corresponding pseudonyms were linked to barcode identifiers. The acceptor blocks consisted of a grid of 11 × 17 tissue cores per TMA block (measuring 25 × 37 mm), which were designed using TMA Control software (version 3.1 SP1, 3DHistech Kft., Budapest, Hungary). For each tumor tissue core, an overlay of the digital cross-sectional image with predefined ROIs and the corresponding FFPE donor block was generated. Manual adjustments were required to ensure precise alignment. After the ROIs were confirmed on the donor blocks, the transfer process was initiated. Tumor cores were automatically punched out of the donor blocks and transferred into the acceptor blocks. Additionally, eight control tissue cores (consisting of tonsil and placenta tissue) were integrated into each TMA block. The TMA layout data and the corresponding clinicopathological data were then exported.

### Immunohistochemistry

Two-micrometre-thick tissue sections were cut from the TMA blocks using a Leica RM2255 rotary microtome (Leica Biosystems, Nussloch, Germany) and deparaffinised. Antigen retrieval was performed using a citrate buffer solution (pH 6.0, 0.01 mol) for five minutes, followed by a 10-minute incubation in a blocking reagent (EnVision® Flex Peroxidase Blocking Reagent, DAKO, SM801). The TMA slides were then stained using the ready-to-use primary antibodies according to the manufacturer's protocols: Oestrogen receptor protein (ER, monoclonal rabbit anti-human oestrogen receptor α, clone EP1, code IR084, Agilent Technologies); progesterone receptor protein (PgR, monoclonal mouse anti-human progesterone receptor, clone PgR 636, code IR068, Agilent Technologies); HER2 (polyclonal rabbit anti-human c-erbB-2 oncoprotein, code A0485, Agilent Technologies); and Ki-67 (monoclonal mouse anti-human Ki-67 antigen, clone MIB-1, code IR626, Agilent Technologies). For UBA1, antigen retrieval was performed in a citrate buffer (0.01 mol, pH 6.0 for 5 minutes). The recombinant rabbit monoclonal antibody (clone SD08-62; MA5-32402; Thermo Fisher Scientific Inc.) was applied at a dilution of 1:250 µg/ml and incubated for one hour. For the streptavidin-biotin-based peroxidase detection method, secondary antibodies (EnVision® Flex+ Rabbit (LINKER) (DAKO, K8019) or EnVision® Flex+ Mouse (LINKER) (DAKO, K8021)) were incubated for 15 minutes. An EnVision® Flex/HRP peroxidase incubation (DAKO, SM802) was performed for 20 minutes. The chromogen used was EnVision FLEX DAB+ substrate buffer, which was incubated for 10 minutes. The nuclei were then counterstained with haematoxylin. To ensure quality, tissue samples from HER2-positive breast cancers (HER2 score 3+ according to Wolff et al. 22) were used as an external positive control for HER2 staining. Physiological breast tissue was used as a control for ER and PgR staining. Non-neoplastic tonsil and placenta tissue were used as controls for UBA1 staining.

### HALO AI Image Analysis of Digitised TMA-Slides

Immunohistochemically stained TMA slides were digitised using a slide scanner (PANNORAMIC 1000, 3DHistech Kft., Budapest, Hungary) and analysed using HALO AI software (version 3.6.4134, Indica Labs Quantitative Pathology, 8700 Education Place NW, Building B, Albuquerque, NM 87114, USA). A preset nuclei segmentation brightfield classifier was adapted for the stained slides. Three different tissue classifiers were trained using the integrated HALO AI DenseNet AI V2 algorithm by manually annotating tissue classes: One was trained for nuclear markers (ER, PgR and MIB-1); one for the membranous marker HER2; and one for evaluating nuclear and cytoplasmic UBA1 expression. The following tissue classes were annotated: Tumor, stroma, adipose tissue, lymphocytes, and background. A total of 12,518 annotations covering a cumulative area of 126.38 mm² were assigned. The performance of the classifiers was validated using the automated HALO AI validation module. Wrongly misclassified areas were manually adjusted to enhance the classifiers' performance. Validation was considered complete once the performance indicators 'precision' (the ratio of correctly predicted pixels to all predicted positive pixels), 'recall' (the ratio of correctly predicted pixels to all pixels), and 'F1-score' (the weighted average of 'precision' and 'recall') each exceeded an accuracy of > 98%. Antibody expression within TMA cores was evaluated and quantified using the HALO AI Multiplex IHC module, including quantity and staining intensity of immunohistochemical staining and percentage of positive cells.

Specific settings were adjusted: 'Nuclear detection' was set to characterise nuclei precisely, including 'nuclear segmentation' to define nuclear boundaries. The 'minimum' and 'maximum' of 'nuclear optical density' (a quantitative measure of nuclear staining intensity) were defined, as were 'size' and 'roundness', to ensure the accurate inclusion of sufficient valid nuclei. Similar settings were applied to detect the membrane and cytoplasm. Threshold settings were refined to categorise staining intensity (0, 1+, 2+, 3+). H-scores and the percentage of cells expressing the cellular marker per core were automatically calculated by HALO AI. The analysed cores were reviewed by board-certified pathologists. Cores with artefacts or an insufficient amount of tumor content were excluded. Finally, the data for each core were exported as a CSV file for further statistical analysis.

### Statistical Analysis

Statistical analysis was performed using the R programming language (v4.0.3; R Core Team, 2020) and RStudio (v2023.03.0+386; RStudio Team, 2020). Integrated Development for R, RStudio PBC, Boston, MA, USA), as previously described 23. The results were stratified by subcellular localisation, with separate analyses performed for the cytoplasmic and nuclear compartments. Descriptive statistics included percentages and median values. A p-value of less than 0.05 was considered statistically significant. A p-value between 0.05 and 0.15 was interpreted as indicating a statistical trend. The Shapiro-Wilk test was performed to test for the normal distribution of UBA1 expression intensity in the cohort. Patients were dichotomised into UBA1 high- and low-expressors using H-Score cutoffs. Cut-off values were calculated using the R package cutpointr 24. Expression was correlated with OS using the log-rank test and Cox regression. Univariable and multivariable analyses within Cox regression models were applied to evaluate the prognostic relevance of the following clinicopathological parameters: age; pT-/pN-status; grading; resection status; lymphatic vessel invasion; blood vessel invasion; perineural invasion; histological subtype; and molecular subtype, as well as UBA1 expression. Parameters showing statistical significance in the univariable analysis were included in the multivariable Cox regression model. The chi-squared test and Fisher's exact test (for small sample sizes) were used to evaluate the association between UBA1 expression and clinicopathological parameters. The Wilcoxon rank-sum test was used to compare non-normally distributed metric variables.

## Results

### Descriptive Statistics - Clinicopathological Parameters

**Figure 1:**
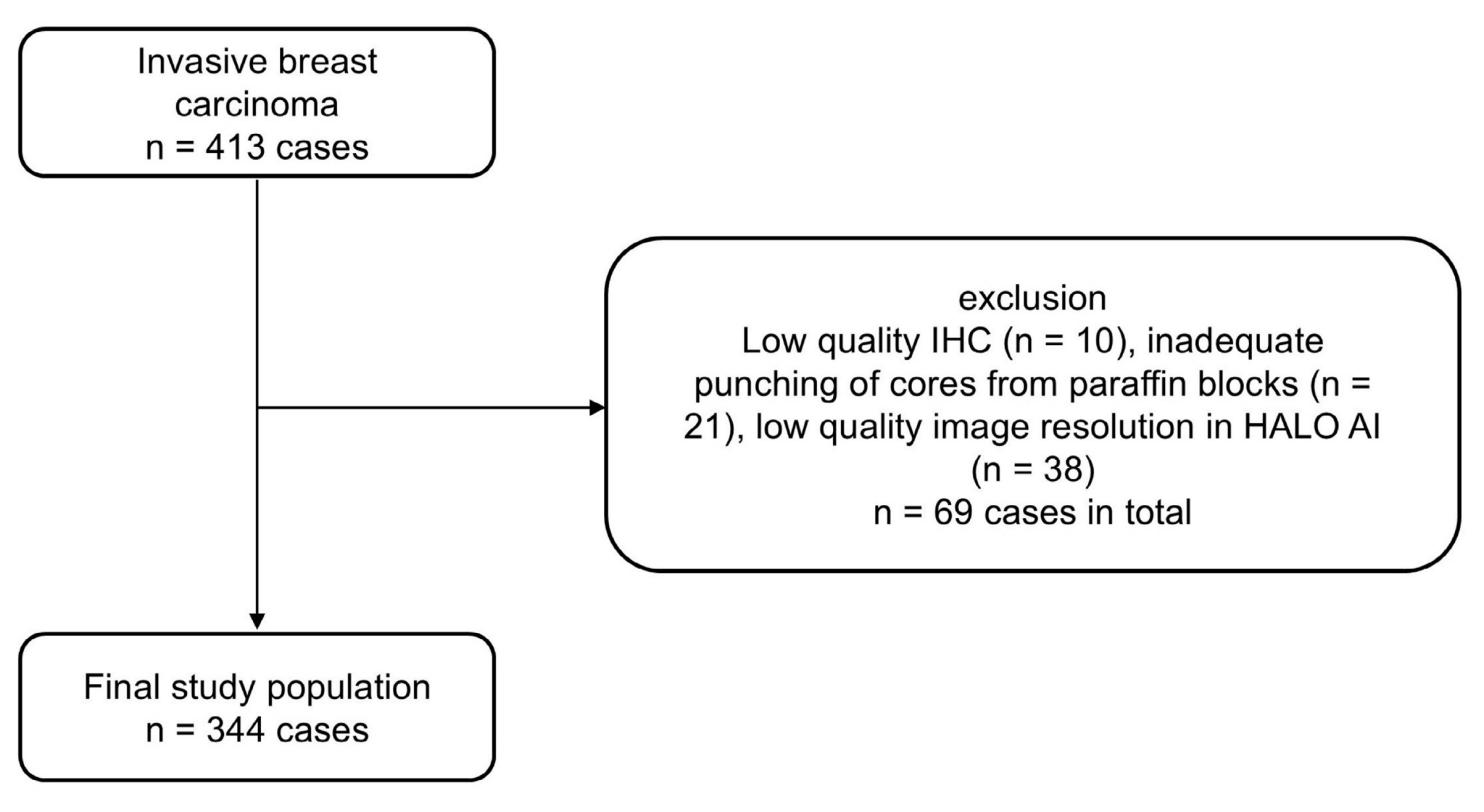
Study Population (CONSORT Flow Diagram).

The study cohort initially comprised 413 tumor-cases. After TMA construction and computer-assisted evaluation, 344 patients were available for statistical analysis (Figure 1).

The median age of patients at diagnosis was 64 years (range: 31-87). The majority of patients were female (n = 342; 99.4%), with two patients being male (0.6%). Of those analysed, 261 patients (76.0%) were alive, while 83 patients (24.0%) had died. The range of OS was 3-157 months, with a median OS of 102 months. The majority of cases were classified as pT1 tumors (n = 199; 57.1%), including pT1b tumors (n = 36; 10.5%) and pT1c tumors (n = 163; 47.4%). No pT1a carcinomas were observed in the cohort. Tumors categorised as pT2 accounted for 36.0% (n = 124) of cases, whereas pT3 (n = 13; 3.8%) and pT4 (n = 8; 2.3%) carcinomas were rare. Tumor-negative local lymph nodes were identified in 226 patients (65.7%), while 105 patients (30.5%) developed local lymph node metastasis (pN1: n = 81 [23.5%]; pN2: n = 14 [4.1%]; pN3: n = 10 [2.9%]). In 13 cases (3.8%), no lymph node resection was performed. Complete surgical resection (R0) was achieved in 315 patients (91.6%). Incomplete resection (R1) was observed in 29 patients (8.4%). Lymphatic vessel invasion (L1) was present in 106 patients (31%), perineural invasion (Pn1) in 19 patients (5.5%), and blood vessel invasion (V1) in seven patients (2%). Tumor grading according to Elston-Ellis 20 yielded 49 G1 (14%), 221 G2 (64%) and 74 G3 (22%) carcinomas. Histologically, the predominant subtype was invasive carcinoma of no special type (NST) (n = 300; 87.2%), followed by invasive lobular carcinoma (n = 35; 10.2%), mucinous carcinoma (n = 6; 1.7%), and rare tubular carcinomas (n = 3; 0.9%). Molecular subtypes were assigned according to established criteria 2,3,21,25,26, demonstrating 144 luminal A (41.9%), 161 luminal B (46.8%), 11 HER2-enriched (3.2%), and 28 (8.1%) triple-negative carcinomas.

### Descriptive Statistics - UBA1

High UBA1 nuclear expression was detected in 102 cases (30%). Low nuclear expression was observed in 242 cases (70%). In the stromal compartment, high nuclear UBA1 expression was present in 121 cases (35%), while low nuclear expression was detected in 223 cases (65%). With respect to cytoplasmic expression in tumor cells, elevated UBA1 expression was observed in 128 cases (37%), while 216 cases (63%) showed low expression. In the stromal compartment, 115 tumors (33%) exhibited high cytoplasmic UBA1 expression, whereas 229 cases (67%) were categorised as low expressors. A detailed summary, along with the results of the bivariate analysis, is provided in Table 1.

For nuclear UBA1 expression, the calculated H-score cutoffs were 131.42 in tumor cells and 82.50 in stromal cells. For cytoplasmic expression, the calculated H-score cutoffs were 108.49 in tumor cells and 99.98 in stromal cells.

### Analysis of the UBA1 Expression Pattern

**Figure 2:**
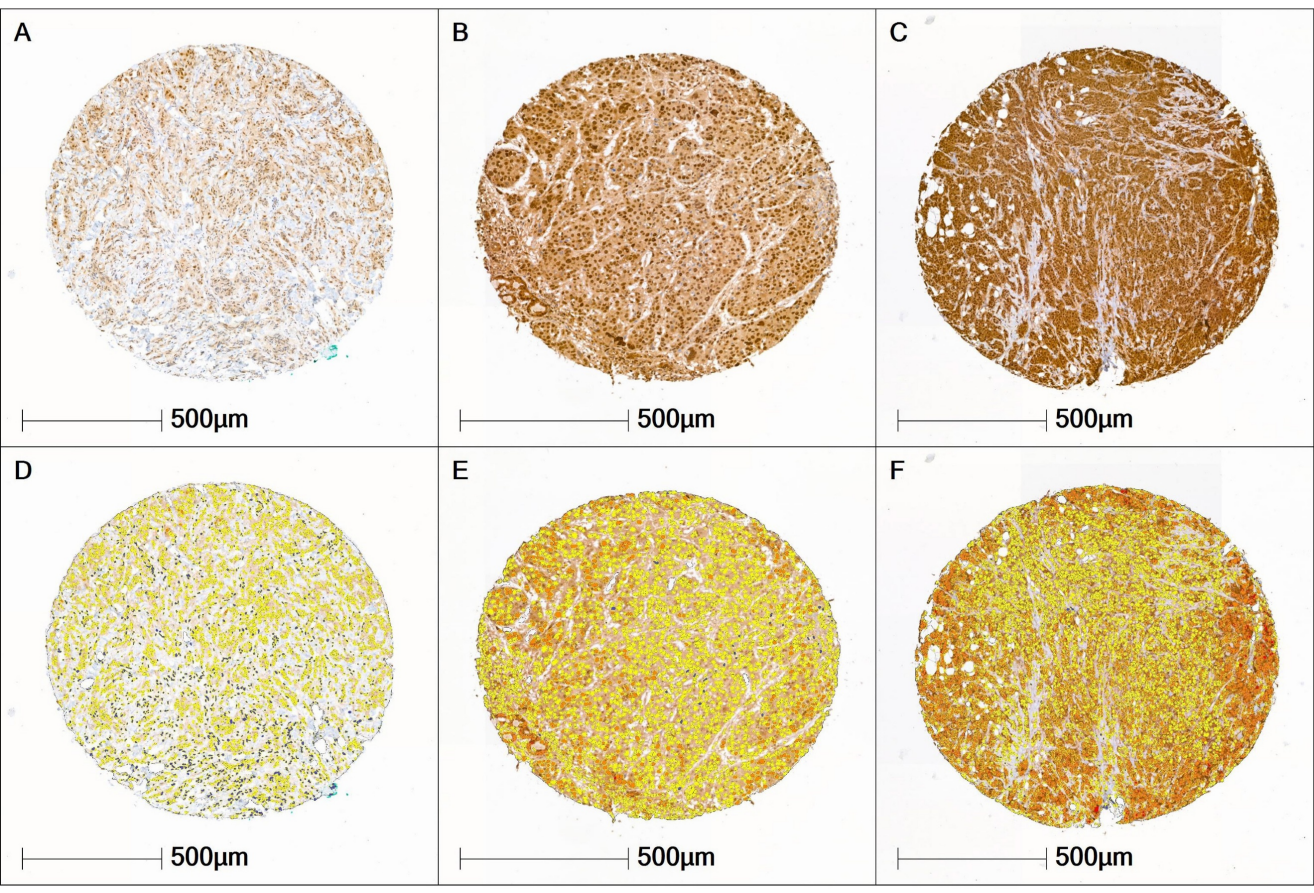
** UBA1 Expression Patterns.** (A, D) Tissue core with low UBA1 expression (tumor H-score 101.4; stroma H-score 99.5. (B, E) Tissue core with moderate UBA1 expression (tumor H-score 118.6; stroma H-score 101.6). (C, F) Tissue core with high UBA1 expression (tumor H-score 156.3; stroma H-score 106.09). Staining intensity is color-coded as follows: blue = negative, yellow = weak, orange = moderate, red = high.

Across the entire cohort, H-scores ranged from 95 to 181 points. Accordingly, the cohort predominantly exhibited a moderate expression pattern in both tumor and stromal cells. UBA1-negative breast carcinomas were not present in the cohort. The Shapiro-Wilk test revealed that UBA1 H-score values were not normally distributed in either the tumor tissue (p < 0.001) or the stromal tissue (p < 0.001), as illustrated in Figure 2.

### Bivariate Analysis

#### Correlation of Nuclear UBA1 Expression and Clinicopathological Parameters

Bivariate analysis revealed statistically significant correlations between nuclear UBA1 expression in tumor cells and age (p = 0.016) and pN-category (p = 0.047). In the stromal compartment, nuclear UBA1 expression was significantly associated with age (p = 0.002), pT-category (p = 0.001), pN-category (p = 0.003), grading (p = 0.004) and intrinsic molecular subtyping (p = 0.028). Furthermore, stromal and tumor-cell UBA1 expression were mutually correlated (p < 0.001) (Table 1).

#### Correlation of Cytoplasmic UBA1 Expression and Clinicopathological Parameters

Bivariate analysis revealed a statistically significant association between cytoplasmic UBA1 expression in tumor cells and the pN-category (p = 0.028). In the stromal compartment, significant associations of cytoplasmic UBA1 expression included age (p = 0.009), pT-category (p = 0.015), pN-category (p = 0.009), tumor grading (p = 0.004) and intrinsic molecular subtyping (p = 0.011).

Additionally, cytoplasmic stromal and tumor cell UBA1 expression were mutually correlated (p < 0.001) (Table 1).

### Log-Rank Test of Overall Survival and UBA1 Expression

**Figure 3:**
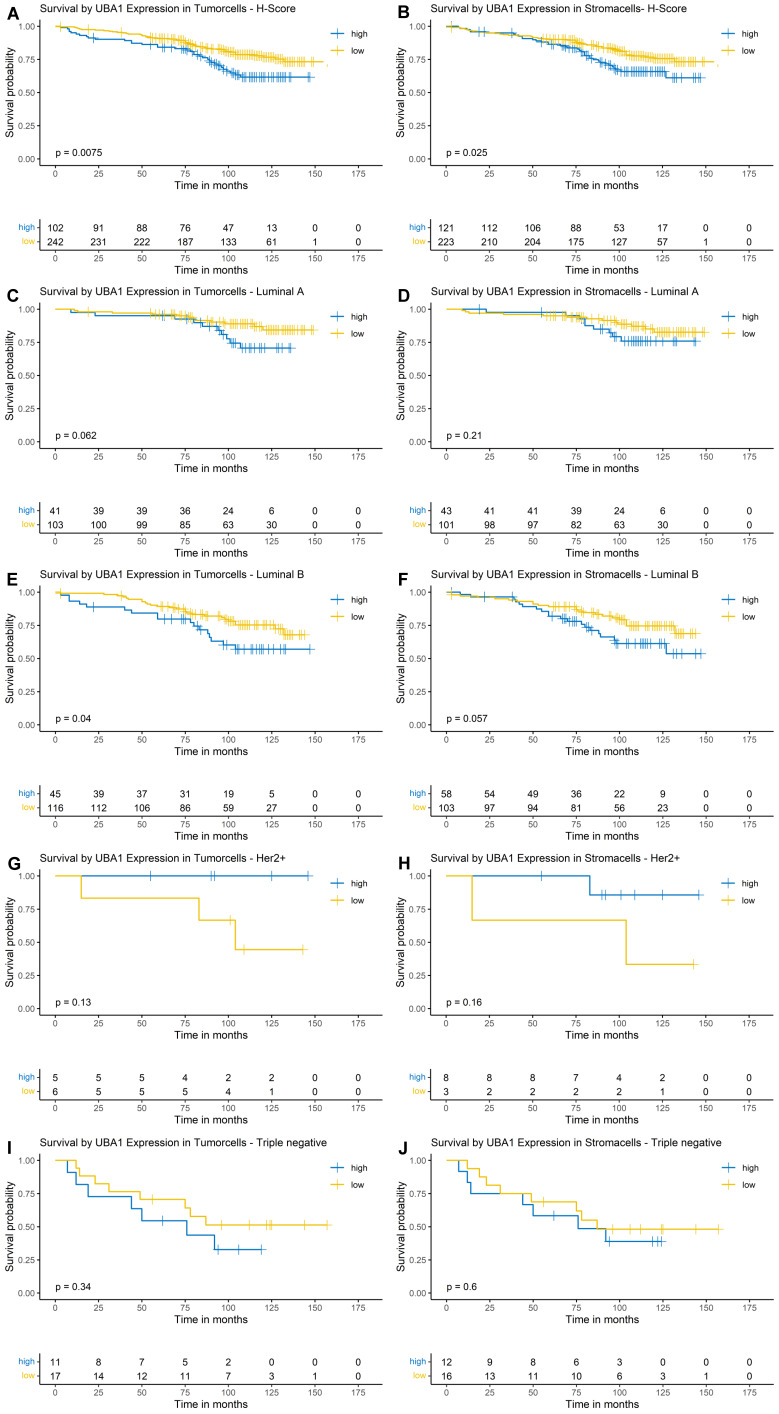
** Kaplan-Meier Survival Analysis by Nuclear UBA1 Expression in Tumor and Stroma.** (A, B) Entire cohort. (C, D) Luminal A subtype. (E, F) Luminal B subtype. (G, H) HER2-enriched subtype. (I + J) Triple-negative subtype.

**Figure 4:**
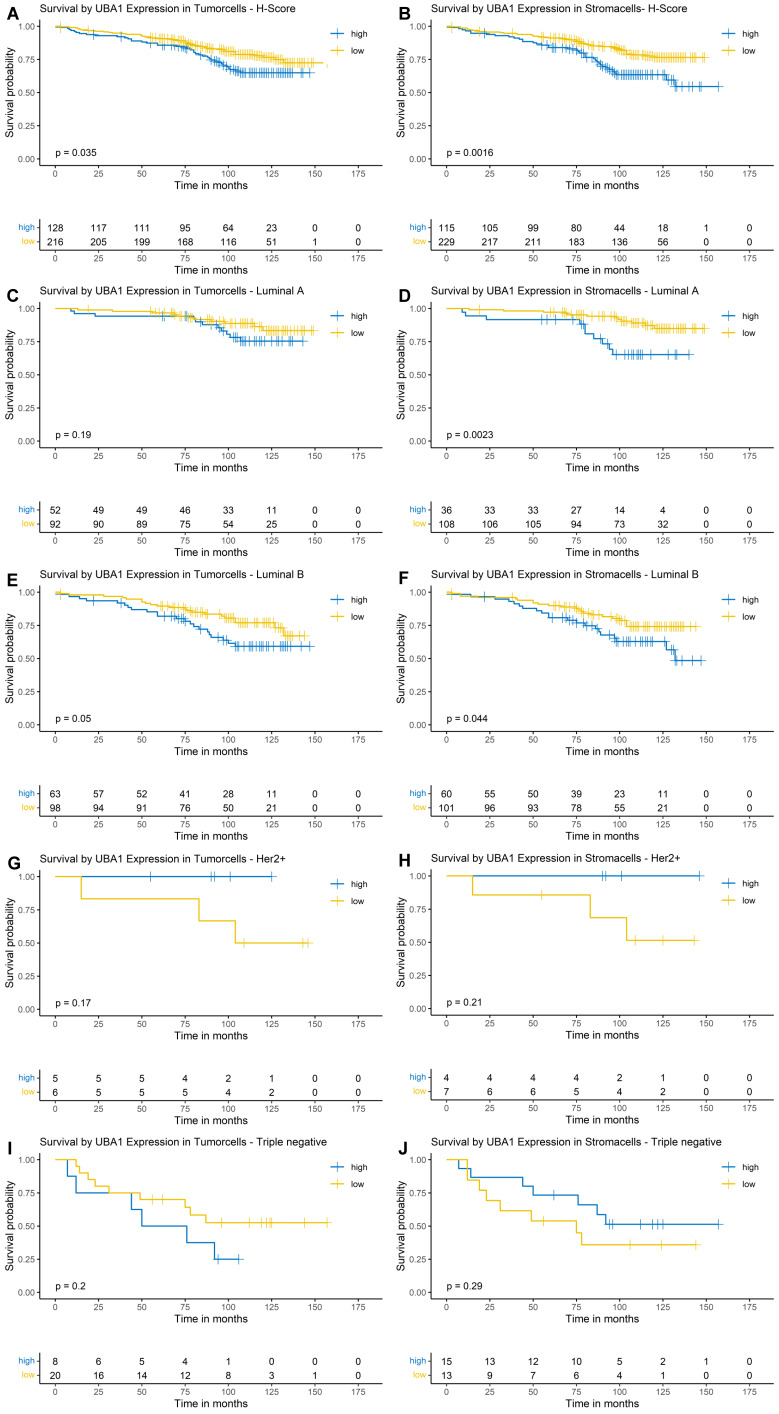
** Kaplan-Meier Survival Analysis by Cytoplasmic UBA1 Expression in Tumor and Stroma.** (A, B) Entire cohort. (C, D) Luminal A subtype. (E, F) Luminal B subtype. (G, H) HER2-enriched subtype. (I + J) Triple-negative subtype.

Using Kaplan-Meier estimation and log-rank testing, a higher nuclear and cytoplasmic UBA1 expression was associated with poorer OS in both tumor (nuclear: p = 0.0075, Figure 3A; cytoplasmic: p = 0.035, Figure 4A) and stromal cells (nuclear: p = 0.025, Figure 3B; cytoplasmic: p = 0.0016, Figure 4B).

#### Nuclear UBA1 Expression

For nuclear expression of UBA1 in tumor cells, the survival function did not fall below a probability of 0.5; therefore, a median OS could not be estimated. The estimated 10-year OS was 62% in the high-expression group, compared to 78% in the low-expression group (Figure 3A). For nuclear UBA1 expression in tumor-associated stroma, the estimated 10-year OS was 65% in the high-expression group versus 76% in the low-expression group (Figure 3B). Along with UBA1 expression in tumor cells, median OS could not be estimated.

In subtype-specific analyses, high nuclear UBA1 expression in tumor cells was significantly associated with poorer OS in Luminal B tumors (p = 0.04, Figure 3E). A trend towards worse OS in cases with high UBA1 expression was also observed in Luminal A tumors (p = 0.062, Figure 3C). In contrast, high tumoral UBA1 expression tended to be associated with a favourable OS in HER2-enriched tumors (p = 0.13, Figure 3G). With respect to the stromal compartment, a trend towards poorer OS associated with high nuclear UBA1 expression was likewise noted in Luminal B tumors (p = 0.057, Figure 3F).

#### Cytoplasmic UBA1 Expression

As the survival function for both the stromal and tumor compartments did not fall below a survival probability of 0.5, a median OS could not be estimated. The estimated 10-year OS was 65% in the high cytoplasmic UBA1 expression group and 77% in the low cytoplasmic UBA1 expression group (Figure 4A). For stromal UBA1 expression, the estimated 10-year OS was 62% in the high-expression group versus 76% in the low-expression group (Figure 4B).

In the subtype-specific analysis, a trend towards poorer OS was observed in Luminal B tumors with higher UBA1 expression levels in tumor cells (p = 0.05; Figure 4E). Analysis of the stromal compartment demonstrated that elevated UBA1 expression in Luminal A and Luminal B tumors was significantly associated with poorer OS (Luminal A: p = 0.0023; Luminal B: p = 0.044). No significant associations or trends were observed in TNBCs and HER2-enriched tumors, although the small number of cases in these subgroups should be taken into consideration.

### Cox Regression of Overall Survival and UBA1 Expression

#### Nuclear UBA Expression

In univariable Cox regression analysis, UBA1 nuclear expression in both tumor cells (p = 0.01) and stromal cells (p = 0.029) was found to have a significant prognostic impact, as were age (p < 0.001), pT-category (p < 0.001), pN-category (p < 0.001), lymphatic vessel invasion (p = 0.023), blood vessel invasion (p = 0.002), perineural invasion (p < 0.001), grading (p < 0.001) and molecular subtype (p < 0.001).

In Cox regression models analysed separately for tumor-cell and stromal UBA1 positivity expression in the tumor compartment retained independent prognostic relevance. Low UBA1 expression in tumor cells was independently associated with improved OS (HR = 0.60, 95% CI 0.37-0.97; p = 0.042). The pT-category also had independent prognostic significance in both models (tumor cell model: p = 0.003 overall; stromal model: p = 0.002 overall), particularly in the comparison of pT4 versus pT1 (tumor cell model: HR = 9.57, 95% CI 2.41-38.0; stromal model: HR = 10.90, 95% CI 2.76-43.0). Perineural invasion was a significant adverse factor in both models (tumor cell model: p = 0.049, HR = 2.50, 95% CI 1.05-5.97; stromal model: p = 0.027, HR = 2.80, 95% CI 1.18-6.68). Additionally, multivariable analysis indicated a trend towards an independent prognostic effect of intrinsic molecular subtyping (tumor cell model: p = 0.12; stromal model: p = 0.10). The results of the Cox regression analysis for nuclear UBA1 expression are presented in Table 2.

#### Cytoplasmic UBA Expression

In univariable analysis, cytoplasmic UBA1 expression demonstrated prognostic significance in both tumor (p = 0.038) and stroma (p = 0.002) as well as age (p < 0.001), pT-category (p < 0.001), pN-category (p < 0.001), lymphatic invasion (p = 0.023), blood vessel invasion (p = 0.002), perineural invasion (p < 0.001), grading (p < 0.001) and molecular subtype (p < 0.001).

In multivariable analysis, low tumor cell UBA1 expression remained independently associated with improved OS (HR = 0.52, 95% CI 0.32-0.84; p = 0.007). The pT-category and Pn-stadium retained independent prognostic effect in both models (pT-stadium: tumor model [p = 0.001]; stromal model [p = 0.002]; Pn-stadium: tumor model [p = 0.028; HR = 2.71, 95% CI 1.16-6.35]; stromal model [p = 0.047; HR = 2.56, 95% CI 1.05-6.21]). For pT-status, the effect was particularly driven by the comparison between pT4 and pT1 stadium (tumor model: HR = 10.7, 95% CI 2.75-41.8; stromal model: HR = 11.0, 95% CI 2.84-42.2). Results of the Cox Regression analysis for the cytoplasmic compartment are presented in Table 3.

## Discussion

UBA1 is expressed in the nucleus and cytoplasm of eukaryotic cells and plays a pivotal role in protein turnover, DNA damage responses and the maintenance of cellular homeostasis 7-9,27. Previous studies have associated UBA1 with cancer progression, including in small-cell lung cancer 28 and hepatocellular carcinoma 29. In haematological malignancies, UBA1 has also been identified as a potential target 12,13,30. With regard to breast cancer, UBA1 has been proposed as a potential target in TNBC cell lines 14. However, the role of UBA1 protein expression across the intrinsic molecular subtypes of breast cancer remains poorly characterised. Therefore, an AI-assisted approach was employed to quantify UBA1 protein expression in tissue microarrays comprising 344 breast cancer specimens.

Log-rank test survival analyses revealed that elevated UBA1 expression in both tumor cell and stromal compartments, at nuclear and cytoplasmic levels, was associated with poorer OS. Subtype-specific analyses revealed that high stromal UBA1 expression, particularly within the cytoplasmic compartment, significantly correlated with poorer OS in Luminal A and Luminal B tumors. Moreover, increased nuclear UBA1 expression in tumor cells was significantly associated with an adverse outcome in the Luminal B subtype. These findings extend previous mRNA-based observations linking higher UBA1 expression levels to poorer OS in hormone receptor-positive breast cancer 15, and further emphasise the contribution of the tumor microenvironment (TME) to disease progression in hormone-dependent carcinomas.

Consistent with these observations, univariable Cox regression analyses revealed similar results, showing a significant negative impact of high nuclear and cytoplasmic UBA1 expression on OS in both tumor cells and the stromal compartment. In multivariable Cox regression models, both nuclear and cytoplasmic UBA1 expression in tumor cells retained independent prognostic significance. In contrast, the prognostic effect of stromal UBA1 expression was confined to univariable analysis, suggesting partial attenuation after adjustment for established clinicopathological confounders. Nevertheless, elevated stromal UBA1 expression was associated with a broader spectrum of adverse clinical and clinicopathological parameters across both subcellular compartments, with particularly pronounced effects observed within the cytoplasmic compartment. Overall, these findings suggest that cytoplasmic UBA1-related signalling mechanisms may play a prominent role in breast cancer development and progression. When interpreting the subtype-specific results presented herein, the small number of cases within the HER2-enriched subgroup (n = 11) and the TNBC subgroup (n = 28) must be taken into account.

From a mechanistic perspective, high stromal UBA1 expression can be considered a surrogate marker of a proteostasis-active and pro-inflammatory TME. As the gatekeeper of ubiquitination, UBA1 has been implicated in key signalling pathways, including the nuclear factor kappa B (NF-κB) signalling pathway 31. NF-κB plays a pivotal role as a transcription factor in regulating inflammation, apoptosis, and tumorigenesis 32. Dysregulation of ubiquitination has been shown to impair NF-κB activity, promoting proinflammatory cytokine-induced cell death 33. In this context, increased UBA1 expression may potentiate NF-κB-linked inflammatory circuits, stress resilience and secretory activity in stromal cell types (e.g. cancer-associated fibroblasts, endothelial cells and immune cells). This supports the promotion of extracellular matrix remodelling, angiogenesis, immune evasion and ultimately tumor progression.

The strong, pronounced impact of stromal UBA1 expression, together with its correlation to adverse clinicopathological features (including advanced age, a higher pT-/pN-category, grade 3 and aggressive subtypes), is consistent with a TME-driven contribution to tumor aggressiveness. Cancer-associated fibroblasts (CAFs) comprise a heterogeneous population of cells within the tumor-associated stroma. They are known to be key modulators of the TME, resulting in tumor progression 34. CAFs have been shown to influence hormone receptor activity in breast cancer, thereby modulating the efficacy of endocrine therapies in luminal breast cancer 35. Consequently, luminal tumors may be particularly dependent on the stromal environment. Elevated stromal UBA1 levels may promote dysregulated, pro-inflammatory CAF function, thereby reinforcing immunosuppression and facilitating tumor progression.

In TNBC, where immune activity can vary between "hot" (immune-active) and "cold" (immune-inactive) states, the effects of immunomodulation are likely to be more heterogeneous. Immune-active tumors, characterised by dense infiltration of immune cells, may benefit from checkpoint blockade (ICB). In contrast, immune-cold tumors, which lack substantial immune cell infiltration, are more challenging to treat and are associated with a poorer prognosis due to the absence of pre-existing immune response mechanisms 36,37. Nevertheless, UBA1 expression within the tumor cell compartment demonstrated retained independent prognostic significance in both the cytoplasmic and nuclear compartments in multivariate analysis, thereby complementing previous findings reported by Feng et al. 15. While our analyses highlight the previously underappreciated contribution of the stroma, the biological and prognostic impact of tumor cell UBA1 expression remains a critical determinant of breast cancer biology. Further delineation of the prognostic and biological relevance of UBA1 across distinct molecular subtypes requires future studies in larger, chemotherapy-naïve cohorts.

Following the development of the first UBA1-specific inhibitor, TAK-243, UBA1 has emerged as a promising therapeutic target 38. Pharmacological UBA1 inhibition by TAK-243 has demonstrated anti-tumor activity in preclinical models of acute myeloid leukaemia (AML) 30 and small cell lung cancer 28, potentially exerting synergistic effects when combined with DNA damage response agents. More recently, potential synergistic effects between UBA1 inhibitors and poly(ADP-ribose) polymerase (PARP) inhibitors have been reported in ovarian cancer and TNBC cell lines 39. Unlike proteasome inhibitors, selective UBA1 inhibition may offer a more favourable toxicity profile 40,41. Furthermore, the literature suggests that UBA1-centred signalling pathways (e.g. the UBA1-STUB1 axis) contribute to immune evasion and resistance to ICB 42. High UBA1 expression has been identified as a strong predictor of therapy resistance and poor survival in cohorts treated with ICB.

Interestingly, antitumor efficacy was enhanced by the synergistic combination of UBA1 inhibition and ICB in preclinical models 42. If this is validated prospectively, patients with high stromal UBA1 expression may particularly benefit from UPS-directed therapeutic strategies, either as monotherapy or in combination with chemotherapy or immunotherapy. As our results expand knowledge of the prognostic relevance of UBA1 in breast cancer, further evaluation of UBA1-targeted therapy inhibitors in breast cancer is warranted, ideally within prospective multicentre trials.

However, certain limitations of the present study should be acknowledged. Firstly, the data were derived from a single-centre cohort, which may have introduced selection bias and limited the generalisability of the findings. Second, potential sources of technical variability arising from TMA preparation and AI-assisted image analysis cannot be fully excluded. Future multicentre, prospective validation studies incorporating orthogonal measurements of CAF/NF-κB activity and curated immune/stromal signatures are warranted to substantiate the proposed mechanisms and clarify the potential therapeutic benefit of UBA1-targeted strategies.

## Conclusion

This study identified UBA1 protein expression in tumor cells as an independent prognostic factor in breast cancer. Elevated UBA1 expression in both tumor and stromal compartments was significantly associated with poor clinical outcomes, particularly in Luminal subtypes. These findings supplement and extend the previously reported mRNA-based results of Feng et al. 15, and emphasise the prognostic relevance of UBA1 expression at the protein level. Given its prognostic significance, further investigation into the potential therapeutic value of UBA1 appears warranted to enhance personalised treatment strategies in breast cancer.

## Figures and Tables

**Table 1:** Clinicopathological Characteristics and Bivariate analysis

Variable	Absolute	UBA1 Expression in tumor cells (nucleus)			UBA1 Expression in stromal cells (nucleus)			UBA1 Expression in tumor cells (cytoplasm)			UBA1 Expression in stromal cells (cytoplasm)		
	N = 344^1^	high N = 102^1^	low N = 242^1^	p-value*^2^*	high N = 121^1^	low N = 223^1^	p-value*^2^*	high N = 128^1^	low N = 216^1^	p-value*^2^*	high N = 115^1^	low N = 229^1^	p-value*^2^*
UBA1 Expression - Tumor							**< 0.001**						**< 0.001**
high	-^4^				63 (52%)	39 (17%)					63 (55%)	65 (28%)	
low	-^4^				58 (48%)	184 (83%)					52 (45%)	164 (72%)	
UBA1 Expression - Stroma				**< 0.001**						**< 0.001**			
high	-^4^	63 (62%)	58 (24%)					63 (49%)	52 (24%)				
low	-^4^	39 (38%)	184 (76%)					65 (51%)	164 (76%)				
Sex				> 0.9			> 0.9			0.14			> 0.9
female	342 (99.4%)	102 (100%)	240 (99%)		120 (99%)	222 (100%)		126 (98%)	216 (100%)		114 (99%)	228 (100%)	
male	2 (0.6%)	0 (0%)	2 (0.8%)		1 (0.8%)	1 (0.4%)		2 (1.6%)	0 (0%)		1 (0.9%)	1 (0.4%)	
Age	64 (31-87)	66 (55, 76)	62 (52, 72)	**0.016**	66 (57, 76)	62 (51, 71)	**0.002**	64 (54, 75)	64 (53, 72)	0.4	67 (56, 75)	62 (52, 71)	**0.009**
pT-status				0.053			**0.001**			0.11			**0.015**
pT1b	36 (10.5%)	6 (5.9%)	30 (12%)		6 (5.0%)	30 (13%)		7 (5.5%)	29 (13%)		5 (4.3%)	31 (14%)	
pT1c	163 (47.4%)	51 (50%)	112 (46%)		47 (39%)	116 (52%)		66 (52%)	97 (45%)		50 (43%)	113 (49%)	
pT2	124 (36%)	34 (33%)	90 (37%)		58 (48%)	66 (30%)		45 (35%)	79 (37%)		50 (43%)	74 (32%)	
pT3	13 (3.8%)	6 (5.9%)	7 (2.9%)		6 (5.0%)	7 (3.1%)		7 (5.5%)	6 (2.8%)		6 (5.2%)	7 (3.1%)	
pT4	8 (2.3%)	5 (4.9%)	3 (1.2%)		4 (3.3%)	4 (1.8%)		3 (2.3%)	5 (2.3%)		4 (3.5%)	4 (1.7%)	
pN-status				**0.047**			**0.003**			**0.028**			**0.009**
pN0	226 (65.7%)	59 (58%)	167 (69%)		68 (56%)	158 (71%)		75 (59%)	151 (70%)		67 (58%)	159 (69%)	
pN1	81 (23.5%)	27 (26%)	54 (22%)		32 (26%)	49 (22%)		37 (29%)	44 (20%)		28 (24%)	53 (23%)	
pN2	14 (4.1%)	6 (5.9%)	8 (3.3%)		5 (4.1%)	9 (4.0%)		5 (3.9%)	9 (4.2%)		5 (4.3%)	9 (3.9%)	
pN3	10 (2.9%)	2 (2.0%)	8 (3.3%)		6 (5.0%)	4 (1.8%)		2 (1.6%)	8 (3.7%)		5 (4.3%)	5 (2.2%)	
No lymph node resection	13 (3.8%)	8 (7.8%)	5 (2.1%)		10 (8.3%)	3 (1.3%)		9 (7.0%)	4 (1.9%)		10 (8.7%)	3 (1.3%)	
Perineural invasion (Pn)				0.8			0.9			0.6			0.7
Pn0	325 (94.5%)	96 (94%)	229 (95%)		114 (94%)	211 (95%)		122 (95%)	203 (94%)		108 (94%)	217 (95%)	
Pn1	19 (5.5%)	6 (5.9%)	13 (5.4%)		7 (5.8%)	12 (5.4%)		6 (4.7%)	13 (6.0%)		7 (6.1%)	12 (5.2%)	
Lymphatic vessel invasion (L)				0.2			0.2			0.5			0.3
L0	238 (69%)	66 (65%)	172 (71%)		78 (64%)	160 (72%)		86 (67%)	152 (70%)		75 (65%)	163 (71%)	
L1	106 (31%)	36 (35%)	70 (29%)		43 (36%)	63 (28%)		42 (33%)	64 (30%)		40 (35%)	66 (29%)	
Blood vessel invasion (V)				0.2			0.2			0.7			0.7
V0	337 (98%)	98 (96%)	239 (99%)		117 (97%)	220 (99%)		125 (98%)	212 (98%)		112 (97%)	225 (98%)	
V1	7 (2.0%)	4 (3.9%)	3 (1.2%)		4 (3.3%)	3 (1.3%)		3 (2.3%)	4 (1.9%)		3 (2.6%)	4 (1.7%)	
Grading				0.5			**0.004**			0.4			**0.004**
G1	49 (14%)	13 (13%)	36 (15%)		13 (11%)	36 (16%)		15 (12%)	34 (16%)		11 (9.6%)	38 (17%)	
G2	221 (64%)	63 (62%)	158 (65%)		70 (58%)	151 (68%)		81 (63%)	140 (65%)		68 (59%)	153 (67%)	
G3	74 (22%)	26 (25%)	48 (20%)		38 (31%)	36 (16%)		32 (25%)	42 (19%)		36 (31%)	38 (17%)	
Residual Disease				0.6			>0.9			0.5			0.3
R0	315 (91.6%)	92 (90%)	223 (92%)		111 (92%)	204 (91%)		119 (93%)	196 (91%)		108 (94%)	207 (90%)	
R1	29 (8.4%)	10 (9.8%)	19 (7.9%)		10 (8.3%)	19 (8.5%)		9 (7.0%)	20 (9.3%)		7 (6.1%)	22 (9.6%)	
Histology				0.6			0.8			0.6			0.7
Invasive breast carcinoma NST	300 (87.2%)	92 (90%)	208 (86%)		105 (87%)	195 (87%)		115 (90%)	185 (86%)		102 (89%)	198 (86%)	
Invasive lobular carcinoma	35 (10.2%)	8 (7.8%)	27 (11%)		14 (12%)	21 (9.4%)		11 (8.6%)	24 (11%)		12 (10%)	23 (10%)	
Mucinous carcinoma	6 (1.7%)	2 (2.0%)	4 (1.7%)		1 (0.8%)	5 (2.2%)		2 (1.6%)	4 (1.9%)		1 (0.9%)	5 (2.2%)	
Tubular carcinoma	3 (0.9%)	0 (0%)	3 (1.2%)		1 (0.8%)	2 (0.9%)		0 (0%)	3 (1.4%)		0 (0%)	3 (1.3%)	
Molecular Subtype				0.4			**0.028**			0.7			**0.011**
HER2-enriched	11 (3.2%)	5 (4.9%)	6 (2.5%)		8 (6.6%)	3 (1.3%)		5 (3.9%)	6 (2.8%)		4 (3.5%)	7 (3.1%)	
Luminal A	144 (41.9%)	41 (40%)	103 (43%)		43 (36%)	101 (45%)		52 (41%)	92 (43%)		36 (31%)	108 (47%)	
Luminal B	161 (46.8%)	45 (44%)	116 (48%)		58 (48%)	103 (46%)		63 (49%)	98 (45%)		60 (52%)	101 (44%)	
Triple negative	28 (8.1%)	11 (11%)	17 (7.0%)		12 (9.9%)	16 (7.2%)		8 (6.3%)	20 (9.3%)		15 (13%)	13 (5.7%)	
Survival status													
Alive	261 (76%)												
Dead	83 (24%)												
Survival in months	102 (3-157)^3^												

*^1^*n (%); Median (Q1, Q3) *^2^*Pearson's Chi-squared test; Fisher's exact test; Wilcoxon rank sum test ^3^Median (Min-Max) ^4^Absolute counts for UBA1 high/low expression were not provided, as UBA1 quantification was performed separately for nuclear and cytoplasmic expression in tumor and stromal compartments

**Table 2:** Cox Regression of Nuclear UBA1 Expression

Characteristic	Absolute	Univariable				Multivariable - Tumor				Multivariable - Stroma			
	N = 344*^1^*	N	HR*^2^*	95% CI*^2^*	p-value	N	HR*^2^*	95% CI*^2^*	p-value	N	HR*^2^*	95% CI*^2^*	p-value
UBA1 Expression - Tumor		344			0.010	344			0.042				
high	102		—	—			—	—					
low	242		0.55	0.36, 0.86			0.60	0.37, 0.97					
UBA1 Expression - Stroma		344			0.029					344			0.2
high	121		—	—							—	—	
low	223		0.61	0.40, 0.94							0.71	0.44, 1.14	
Sex		344			0.053								
female	342		—	—									
male	2		5.89	1.44, 24.0									
Age	64 (53, 73)	344	1.07	1.05, 1.09	**<0.001**								
pT-status		344			**<0.001**	344			**0.003**	344			**0.002**
pT1b	36		—	—			—	—			—	—	
pT1c	163		0.88	0.38, 2.01			0.69	0.29, 1.63			0.72	0.30, 1.69	
pT2	124		1.61	0.71, 3.63			1.05	0.45, 2.45			1.00	0.42, 2.39	
pT3	13		4.00	1.40, 11.5			1.90	0.55, 6.48			2.00	0.59, 6.82	
pT4	8		37.3	12.4, 113			9.57	2.41, 38.0			10.9	2.76, 43.0	
pN-status		344			**<0.001**	344			0.9	344			0.9
pN0	226		—	—			—	—			—	—	
pN1	81		1.35	0.79, 2.31			1.31	0.66, 2.62			1.35	0.67, 2.69	
pN2	14		4.63	2.25, 9.55			1.74	0.58, 5.20			1.73	0.59, 5.11	
pN3	10		6.10	2.85, 13.1			1.83	0.52, 6.45			1.38	0.39, 4.90	
No lymph node resection	13		3.14	1.24, 7.98			1.13	0.38, 3.36			1.20	0.40, 3.61	
Perineural invasion (Pn)		344			**<0.001**	344			**0.049**	344			**0.027**
Pn0	325		—	—			—	—			—	—	
Pn1	19		4.80	2.59, 8.90			2.50	1.05, 5.97			2.80	1.18, 6.68	
Lymphatic vessel invasion (L)		344			0.023	344			0.5	344			0.6
L0	238		—	—			—	—			—	—	
L1	106		1.67	1.08, 2.59			0.80	0.40, 1.61			0.82	0.41, 1.64	
Blood vessel invasion (V)		344			**0.002**	344			0.9	344			>0.9
V0	337		—	—			—	—			—	—	
V1	7		6.17	2.48, 15.3			0.89	0.25, 3.14			1.00	0.29, 3.48	
Grading		344			**<0.001**	344			0.3	344			0.4
G1	49		—	—			—	—			—	—	
G2	221		2.77	1.00, 7.67			1.96	0.68, 5.62			1.93	0.67, 5.57	
G3	74		5.32	1.87, 15.1			2.24	0.69, 7.26			2.09	0.64, 6.82	
Residual Disease		344			0.8								
R0	315		—	—									
R1	29		0.88	0.38, 2.03									
Histology		344			0.5								
Invasive breast carcinoma NST	300		—	—									
Invasive lobular carcinoma	35		1.29	0.66, 2.50									
Mucinous carcinoma	6		1.34	0.33, 5.46									
Tubular Carcinoma	3		0.00	0.00, Inf									
Molecular Subtype		344			**<0.001**	344			0.12	344			0.10
HER2-enriched	11		—	—			—	—			—	—	
Luminal A	144		0.53	0.16, 1.76			0.71	0.20, 2.58			0.69	0.19, 2.54	
Luminal B	161		1.05	0.33, 3.39			1.10	0.33, 3.71			1.08	0.32, 3.64	
Triple negative	28		2.71	0.78, 9.35			1.92	0.52, 7.07			1.95	0.53, 7.22	

*^1^*n (%); Median (Q1, Q3) *^2^*HR = Hazard Ratio, CI = Confidence Interval

**Table 3:** Cox Regression of Cytoplasmic UBA1 Expression

Characteristic	Absolute	Univariable				Multivariable - Tumor				Multivariable - Stroma			
	N = 344*^1^*	N	HR*^2^*	95% CI*^2^*	p-value	N	HR*^2^*	95% CI*^2^*	p-value	N	HR*^2^*	95% CI*^2^*	p-value
UBA1 Expression - Tumor		344			**0.038**	344			0.007				
high	128		—	—			—	—					
low	216		0.63	0.41, 0.97			0.52	0.32, 0.84					
UBA1 Expression - Stroma		344			**0.002**					344			0.11
high	115		—	—							—	—	
low	229		0.51	0.33, 0.78							0.68	0.43, 1.09	
Sex		344			0.053								
female	342		—	—									
male	2		5.89	1.44, 24.0									
Age	64 (53, 73)	344	1.07	1.05, 1.09	**<0.001**								
pT-status		344			**<0.001**	344			**0.001**	344			**0.002**
pT1b	36		—	—			—	—			—	—	
pT1c	163		0.88	0.38, 2.01			0.65	0.27, 1.53			0.73	0.31, 1.73	
pT2	124		1.61	0.71, 3.63			0.99	0.42, 2.32			1.04	0.44, 2.45	
pT3	13		4.00	1.40, 11.5			1.79	0.52, 6.10			2.05	0.60, 7.02	
pT4	8		37.3	12.4, 113			10.7	2.75, 41.8			11.0	2.84, 42.2	
pN-status		344			**<0.001**	344			0.8	344			0.8
pN0	226		—	—			—	—			—	—	
pN1	81		1.35	0.79, 2.31			1.35	0.68, 2.70			1.34	0.67, 2.67	
pN2	14		4.63	2.25, 9.55			1.81	0.61, 5.37			1.85	0.64, 5.35	
pN3	10		6.10	2.85, 13.1			1.83	0.53, 6.37			1.41	0.39, 5.03	
No lymph node resection	13		3.14	1.24, 7.98			1.01	0.33, 3.07			1.25	0.42, 3.68	
Perineural invasion (Pn)		344			**<0.001**	344			**0.028**	344			**0.047**
Pn0	325		—	—			—	—			—	—	
Pn1	19		4.80	2.59, 8.90			2.71	1.16, 6.35			2.56	1.05, 6.21	
Lymphatic vessel invasion (L)		344			0.023	344			0.5	344			0.6
L0	238		—	—			—	—			—	—	
L1	106		1.67	1.08, 2.59			0.79	0.39, 1.58			0.84	0.42, 1.67	
Blood vessel invasion (V)		344			**0.002**	344			>0.9	344			>0.9
V0	337		—	—			—	—			—	—	
V1	7		6.17	2.48, 15.3			0.99	0.29, 3.38			0.99	0.29, 3.31	
Grading		344			**<0.001**	344			0.4	344			0.5
G1	49		—	—			—	—			—	—	
G2	221		2.77	1.00, 7.67			1.93	0.67, 5.53			1.83	0.63, 5.28	
G3	74		5.32	1.87, 15.1			2.18	0.67, 7.02			2.00	0.61, 6.56	
Residual Disease		344			0.8								
R0	315		—	—									
R1	29		0.88	0.38, 2.03									
Histology		344			0.5								
Invasive breast carcinoma NST	300		—	—									
Invasive lobular carcinoma	35		1.29	0.66, 2.50									
Mucinous carcinoma	6		1.34	0.33, 5.46									
Tubular Carcinoma	3		0.00	0.00, Inf									
Molecular Subtype		344			**<0.001**	344			0.057	344			0.15
HER2-enriched	11		—	—			—	—			—	—	
Luminal A	144		0.53	0.16, 1.76			0.62	0.17, 2.30			0.67	0.18, 2.44	
Luminal B	161		1.05	0.33, 3.39			0.97	0.28, 3.28			0.98	0.29, 3.31	
Triple negative	28		2.71	0.78, 9.35			1.98	0.54, 7.34			1.74	0.47, 6.43	

*^1^*n (%); Median (Q1, Q3) *^2^*HR = Hazard Ratio, CI = Confidence Interval

## Abbreviations

UBA1: Ubiquitin like modifier activating enzyme 1
H&E: Haematoxylin and Eosin
ROI: Region of interest
TMA: Tissue Microarray
ER: Oestrogen Receptor
PgR: UPe Receptor
HER2: Human epidermal growth factor receptor 2
Ki-67: Kiel Antigen Nr. 67
AI: Artificial intelligence
UPS: Ubiquitin-proteasome system
TNBC: Triple-negative breast cancer
CRISPR/Cas: Clustered Regularly Interspaced Short Palindromic Repeats/CRISPR-associated protein
HR: Hazard ratio
CI: Confidence Interval
AML: acute myeloid leukaemia
WHO: World Health Organization
UICC: Union Internationale Contre le Cancer
OS: Overall survival
ICB: Immune Checkpoint blockade
CAFs: Cancer-associated fibroblasts
NF-kB: Nuclear factor kappa B
TME: Tumor microenvironment
FFPE: Formalin-fixed, paraffin-embedded
NST: No Special Type
SEER: Surveillance, Epidemiology, and End Results
